# The stretch-shortening cycle (SSC) revisited: residual force enhancement contributes to increased performance during fast SSCs of human m. adductor pollicis

**DOI:** 10.14814/phy2.12401

**Published:** 2015-05-13

**Authors:** Wolfgang Seiberl, Geoffrey A Power, Walter Herzog, Daniel Hahn

**Affiliations:** 1 Department of Biomechanics in Sports, Faculty of Sport and Health Sciences, Technische Universität MünchenMunich, Germany; 2 Human Performance Laboratory, Faculty of Kinesiology, University of CalgaryCalgary, Canada; 3 Human Movement Science, Faculty of Sports Science, Ruhr-Universität BochumBochum, Germany

**Keywords:** Concentric, eccentric, electrical stimulation, force depression, force enhancement, force redevelopment, muscle, potentiation, thumb

## Abstract

The stretch-shortening cycle (SSC) occurs in most everyday movements, and is thought to provoke a performance enhancement of the musculoskeletal system. However, mechanisms of this performance enhancement remain a matter of debate. One proposed mechanism is associated with a stretch-induced increase in steady-state force, referred to as residual force enhancement (RFE). As yet, direct evidence relating RFE to increased force/work during SSCs is missing. Therefore, forces of electrically stimulated m. adductor pollicis (*n* = 14 subjects) were measured during and after pure stretch, pure shortening, and stretch-shortening contractions with varying shortening amplitudes. Active stretch (30°, *ω *= 161 ± 6°s^−1^) caused significant RFE (16%, *P* < 0.01), whereas active shortening (10°, 20°, and 30°; *ω *= 103 ± 3°s^−1^, 152 ± 5°s^−1^, and 170 ± 5°s^−1^) resulted in significant force depression (9–15%, *P* < 0.01). In contrast, after SSCs (that is when active stretch preceded active shortening) no force depression was found. Indeed for our specific case in which the shortening amplitude was only 1/3 of the lengthening amplitude, there was a remnant RFE (10%, *P* < 0.01) following the active shortening. This result indicates that the RFE generated during lengthening affected force depression when active lengthening was followed by active shortening. As conventional explanations, such as the storage and release of elastic energy, cannot explain the enhanced steady-state force after SSCs, it appears that the stretch-induced RFE is not immediately abolished during shortening and contributes to the increased force and work during SSCs.

## Introduction

Stretch-shortening cycles (SSC) occur in most natural movements. During a SSC, a lengthening contraction is immediately followed by a shortening contraction that may be shortly delayed due to a brief transition phase (Komi and Gollhofer 1997; Komi 2000). In SSCs the force/work performed during the shortening phase is typically greater than that observed when shortening is not preceded by active stretching. This increase in performance has been observed in isolated muscles, in situ animal preparations (Cavagna et al. 1968; Gregor et al. 1988), and during maximal voluntary contractions of human muscles (Cavagna et al. 1968; Bosco et al. 1987).

Similar observations on enhanced forces can be made in steady-state isometric forces following an active muscle stretch. Such forces are increased compared to those obtained for purely isometric contractions (Edman 2012). This property of skeletal muscle has been called residual force enhancement (RFE) (Edman et al. 1982). Although the underlying mechanisms are still not entirely understood (Edman 2012; Rassier 2012; Herzog 2014), RFE has been observed consistently, and its manifestation has been described in many research papers since the 1950's. RFE increases with increasing stretch amplitude (Abbott and Aubert 1952; Edman et al. 1978, 1982), is present at all muscle lengths (Rassier et al. 2003; Peterson et al. 2004), and is independent of stretch velocity (Edman et al. 1978; Sugi and Tsuchiya 1988). In contrast, isometric steady-state forces following active shortening are decreased compared to the corresponding purely isometric forces: a property referred to as force depression (FD) (Edman 1976; Lee and Herzog 2003). FD increases with increasing shortening magnitudes (Marechal and Plaghki 1979; Herzog and Leonard 1997) and decreases with increasing shortening speeds (Marechal and Plaghki 1979; Herzog and Leonard 1997), however FD does not appear to be directly related to the speed of shortening, but rather to the force or work during shortening which changes as a function of speed (Herzog et al. 2000). Observations of FD and RFE have been made across all structural levels of muscle, in a variety of muscles, and most importantly in the current context, have also been made for in vivo human muscles activated voluntarily or artificially via electrical stimulation (Lee et al. 1999, 2000; Lee and Herzog 2002; Oskouei and Herzog 2005; Hahn et al. 2010, 2012; Seiberl et al. 2010, 2012; Power et al. 2012, 2013, 2014).

**Figure 1:**
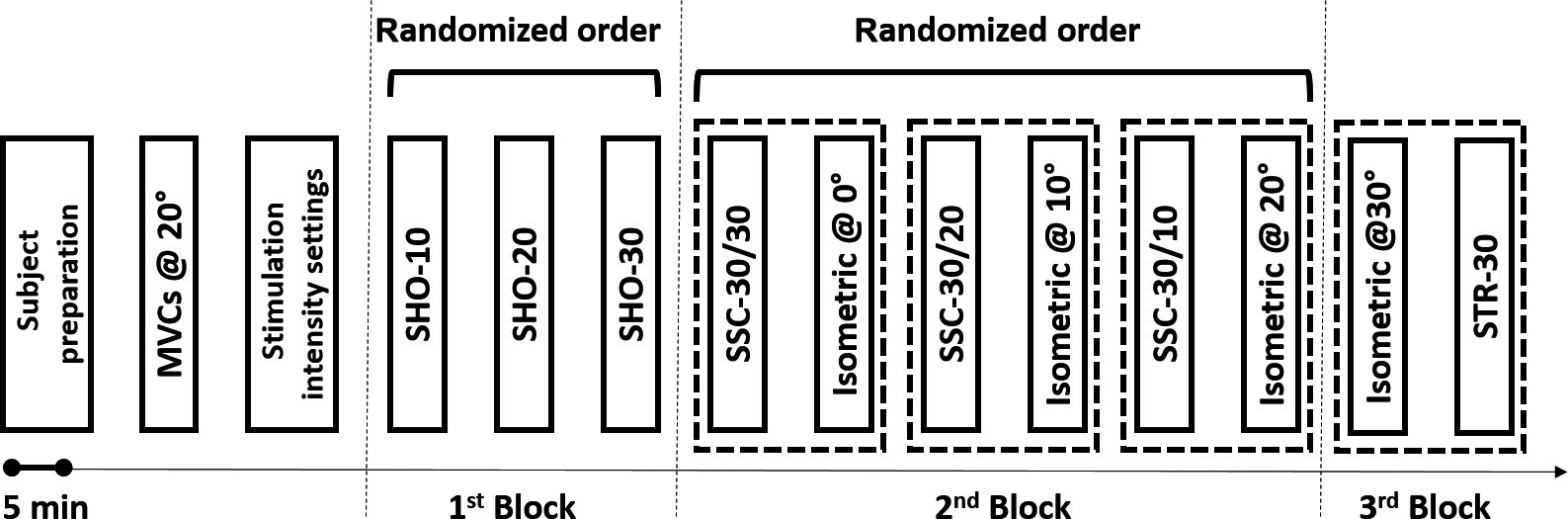
Experimental protocol. After subject preparation, assessment of voluntary maximum force (MVC) at 20° thumb angle, and adjustments of the stimulation intensity (50–60% of MVC), the experiment was started using a pseudorandomized block design. First block: 3 pure shortening contractions beginning at 30° with shortening amplitudes of 10° (SHO-10), 20° (SHO-20) and 30° (SHO-30). Second block: 3 SSCs and corresponding isometric reference contractions at 0°, 10°, and 20°, pairwise randomized. SSCs started at 0° with 30° lengthening. In SSC-30/30, 30° lengthening was followed by 30° shortening ending at 0°. In SSC-30/20 and SSC-30/10, the 30° stretch was followed by 20° and 10° shortening, ending at 10° and 20°, respectively. Third block: pure stretch contraction (STR-30) from 0° to 30° always performed after the isometric reference contraction at 30°.

An active stretch during SSCs produces the same amount of RFE as an active stretch alone. Therefore, it has been speculated that the increased force/work observed in the shortening phase of SSCs might be caused in part by mechanisms associated with RFE (Cavagna et al. 1968). However, work on the cat soleus indicated that the effects of RFE are eliminated quickly by active muscle shortening (Herzog and Leonard 2000; Lee et al. 2001), thus not necessarily supporting this idea. However, despite force depressed states at the end of shortening presented in the work of Lee et al. (2001), enhanced forces (compared to pure concentric contractions) can be observed in the first phase of shortening during SSCs, immediately after lengthening at the beginning of the concentric phase (see figure 1b, Lee et al. 2001).

Aside from mechanisms associated with RFE, three other primary mechanisms have been proposed to contribute to the force/work of muscles in the shortening phase of SSCs: (1) the activation dynamics; (2) contributions of stretch reflexes; and (3) the storage and release of elastic energy (van Ingen Schenau et al. 1997a,b). However, there is a lack of understanding how these different factors might contribute to the enhancement of performance of shortening contractions that are preceded by stretch in general, and specifically the possible role of RFE. Experimental evidence suggests that the activation dynamics, stretch-reflex responses, and the release of stored elastic energy cannot account for the total increase in force/work observed during shortening of SSCs (Ettema et al. 1992; Walshe et al. 1998). Therefore, stretch-induced sarcomeric force enhancement might be the missing link. In this case, associated active and/or passive RFE mechanisms triggered by active stretch of a muscle would be expected to counteract the well accepted influence of FD observed for pure shortening. Accordingly, if RFE-related mechanisms contribute to the increased performance of muscle shortening following stretch, one of the following hypotheses should prove correct: (1) there is RFE in the steady-state isometric phase after a SSC; or (2) FD is reduced following SSCs compared to pure shortening contractions. The purpose of this study was to test these hypotheses.

In order to minimize confounding factors such as the influence of (voluntary) activation, stretch reflexes, or recoil effects of large human muscle-tendon complexes, electrical stimulation of the human m. adductor pollicis was used as a model taking advantage of equipment and methods described previously (Lee and Herzog 2003). As naturally occurring SSC contractions typically follow a stretch-shortening motor program of less than ~250 msec (e.g., drop-jump), we focused on fast muscle actions, characterized by high contraction velocities and small angular displacements. The magnitudes of angular velocity and/or displacement are known to affect RFE and FD, with RFE unaffected by velocity and FD minimal for fast speeds of shortening. In addition, stretch-shortening cycles were performed using a constant stretch amplitude and varying shortening magnitudes to observe the possible decay of RFE contributions to shortening performance with increasing shortening magnitudes.

## Methods

### Participants

Fourteen healthy male subjects (age 28.9 ± 5.8 years; height 180.2 ± 8.0 cm; weight 78.0 ± 10.9 kg) gave free written informed consent prior to participating in this study. All subjects were free of neuromuscular disorders or injury to the left hand. All experimental procedures were approved by the Conjoint Ethics Committee of the University of Calgary.

### Experimental setup

Thumb adduction force and carpometacarpal angular displacement were measured using a custom-designed dynamometer (Lee and Herzog 2002). The left hand was immobilized with a reusable clinical cast (Ezeform, Rehabilitation Division, Smith & Nephew Inc., Germantown, WI) and was secured with two Velcro straps, restricting movement of the wrist and fingers except for the thumb. Subjects sat on an adjustable chair with the forearm slightly abducted and the elbow flexed at 90°. A rotary stepper motor (Model TS42BP10, Parker Hannifin Corp., Cleveland, OH) was connected to an aluminum rod (1.5 cm diameter and 15 cm long) via gears (1:4 gear ratio). The other end of the rod was attached to an auxiliary piece for thumb placement and fixation. The thumb pressed on the auxiliary piece, which was in line with the direction of force measurement obtained through two pairs of calibrated strain gauges (Model CEA-06–125UN-350, Measurement Group, Inc. Raleigh, NC). A lack of slipping of the thumb during range of motion testing ensured alignment of the carpometacarpal joint with the center of rotation of the motor. The hand was slightly externally rotated, thus movement of the thumb was guided toward the third finger. Thumb angle was measured using an analog encoder (Series 03 rotary transducer; Hohner Corp., UK). A digital controller (Model Gemini GT6-L8 Digital stepper driver/controller, Parker Hannifin Corp.) controlled the rod. A 0° reference angle was defined for each subject as the highest degree of adduction possible before the dynamometer arm came in contact with the cast. Thumb angles increased with abduction, ranging from 0 to 30°, thereby ending approximately on the plateau of the force–angle relationship of thumb adduction (de Ruiter et al. 2000).

### Electrical stimulation

Two self-adhering Ag-AgCl surface electrodes (2 × 3 cm) were placed over the ulnar nerve to electrically stimulate the adductor pollicis. The cathode was placed approximately 2 cm proximal to the pisiform bone on the medial wrist, and the anode was placed 2 cm proximal to the cathode. A computer-triggered stimulator (model DS7AH, Digitimer, Welwyn Garden City, Hertfordshire, UK) was used to increase current until further increases failed to produce an increase in peak twitch force amplitude (single 100 *μ*sec square-wave pulses). The last supra-maximal stimulation setting was used to assess voluntary activation during maximum voluntary efforts (MVC) using the interpolated twitch technique (Merton 1954). Tetanic electrical stimulation was performed with an identical setup, device and electrode configuration. Current was increased (50 Hz; square-wave pulses with 100 *μ*sec pulse width) until evoked tetanic force reached a level between 50 to 60% of the participants MVC force.

### Protocol

First, peak twitch force amplitude was determined as mentioned above. Then, subjects performed 1–2 MVCs at a carpometacarpal angle of 20° in order to assess individual peak force (set to 100% MVC) and voluntary activation. Participants were encouraged verbally during all MVCs and were provided with visual feedback of the torque tracing on a computer monitor. Only subjects with voluntary activation >95% were used for further testing.

All contraction types were pseudorandomized in a block design (Fig.1). The first block contained three pure shortening contractions (SHO) always beginning at 30° with shortening amplitude of 10° (SHO-10), 20° (SHO-20), and 30° (SHO-30) randomized. The second block contained three randomized SSCs and the corresponding isometric reference contractions at 0°, 10°, and 20° thumb adduction angle. SSCs always started at 0° with 30° lengthening. In SSC-30/30, 30° lengthening was followed with a 10 ms delay by 30° shortening ending at 0°. In SSC-30/20 and SSC-30/10, the 30° stretch was followed by 20° and 10° shortening, ending at a 10° and 20° thumb adduction angle, respectively. The last block consisted of a pure stretch contraction (STR-30) from 0° to 30° and its isometric reference contraction at 30°. In the second and third block, SSC contractions always preceded the isometric reference contractions, and lengthening contractions were always last to exclude fatigue as a confounding factor. Thus, if at all, any bias due to fatigue would result in an underestimation of the enhanced or the depressed forces. Contractions within blocks one and two where (pairwise) randomized. All dynamic contractions (stretch and shortening) were accelerated with 500°s^−2^ leading to calculated mean velocities of 170 ± 5°/sec, 152 ± 5°/sec, and 103 ± 3°/sec for the 30°, 20°, and 10° shortening amplitudes, respectively, and 161 ± 6°/sec for the 30° lengthening tests. Each contraction lasted ~6.5 sec (2 sec before and 4 sec after the angular displacements). Each trial was separated by a minimum of 5 min rest.

### Data reduction and analysis

**Figure 2:**
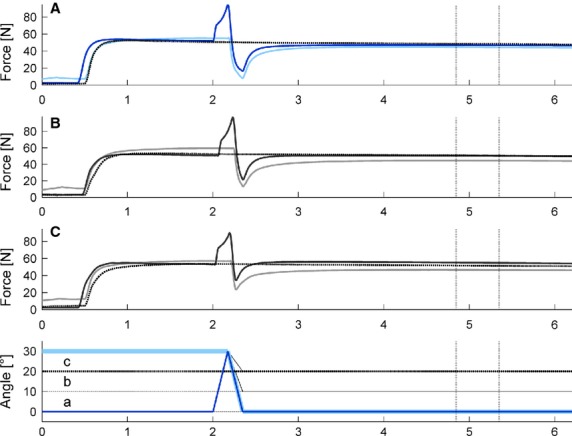
Typical force-time (*n* = 1, filtered with lowpass 10 Hz) and angle-time (reduced schematic illustration) traces of pure shortening (light blue/gray), stretch-shortening (dark blue/gray) and isometric references (dotted black lines) at final thumb angle position 0° (A, a), 10° (B, b), and 20° (C, c). Pure shortening contractions always started at a 30° thumb angle, stretch-shortening always started from 0° with 30° lengthening immediately followed by 30° (a), 20° (b), or 10° (c) of shortening. Steady-state muscle forces were measured between 2.5–3s after end of shortening and at the corresponding times for the isometric reference contractions (vertical lines). Exemplarily highlighted the force-time and angle-time traces of stretch-shortening (dark blue) versus pure shortening (light blue) with shortening amplitude of 30°.

All data were sampled at 2000 Hz and collected via an analog-to-digital converter (PowerLab System 16/35, ADInstruments, Bella Vista, Australia). Force data were filtered (low pass 10 Hz) and peak force (end of lengthening), minimum force (end of shortening), and mean force during a 500 msec time period at 2.5–3 sec after the end of shortening or lengthening, were used for statistical analysis (Fig.2), and were compared to the corresponding isometric reference forces.

In addition to steady-state characteristics after pure shortening and stretch-shortening contractions, the transient period of force recovery following active shortening was analyzed to quantify force redevelopment over time, *F*_Red_(t). Force-time data were fitted using a best-fit double exponential function (equation 1) by means of commercial software MATLAB (R2013b, MathWorks, Inc, Natick, MA): 

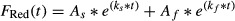
1

**Figure 3:**
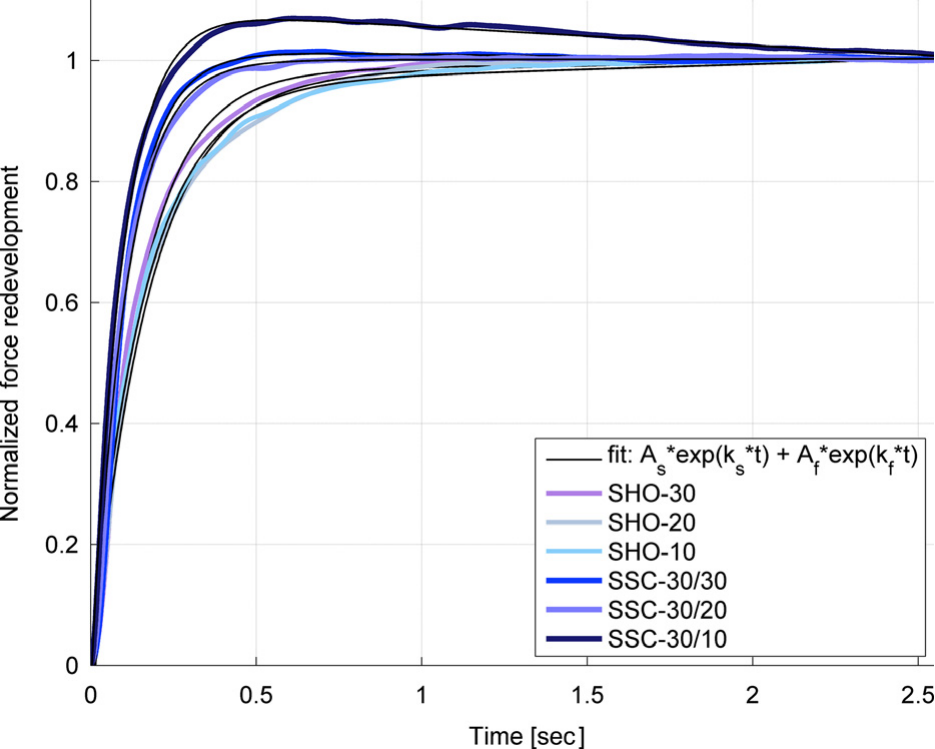
Typical data (*n* = 1, filtered with lowpass 10 Hz) of force redevelopment after pure shortening (SHO-30, SHO-20, SHO-10) and stretch-shortening (SSC-30/30, SSC-30/20, SSC-30/10) contractions and corresponding double exponential fit (black lines, *R*^2^ > 0.99). Force is normalized to the amount of force redevelopment, meaning 0 represents the force at the end of shortening and 1 corresponds to steady-state force 2.5–3 sec after shortening. SSC contractions show higher rates of fast redevelopment (*k*_*f*_) and reduced half-value periods.

For this equation 1 all force-time data were normalized such that 0 force corresponded to the minimum force after shortening, and 1 corresponded to the mean value of force at 2.5–3s after the end of the shortening phase (Fig.3). The amount of force redevelopment during the fast and slow recovery-phase is given by ***A***_***f***_ and ***A***_***s***_, in equation 1, respectively, and the force redevelopment rates are given by ***k***_***f***_ and ***k***_***s***_ for the fast and slow parts of the force-time traces, respectively. To assure the curve fitting parameters represent the measured force redevelopment data accurately, trials that could not be fitted with a quality of *R*² > 0.96 (*n* = 4) were not used for statistics. For further analysis, parameters of equation 1 were pooled according to pure shortening and stretch-shortening trials.

### Statistical analysis

Data were tested for normality (Kolmogorov–Smirnov test) and either repeated measures ANOVA (or nonparametric Friedman tests) with Bonferroni–Holm post hoc comparisons (Students *t*-tests or Wilcoxon test) were used to identify significant differences between parameters of mean force, work and parameters of force redevelopment after pure shortening and stretch-shortening, as well as for comparisons of forces to the corresponding purely isometric reference contractions. Statistical significance was set at *P* < 0.05. Outliers, defined as force values exceeding the respective mean by more than ± 2SD, were excluded from statistical analysis.

## Results

### Baseline

The mean electrically evoked tetanic isometric peak force at 20° was 52.1 ± 9.6N, representing 50.0 ± 5.7% of the initial force achieved during MVCs (104.4 ± 15.6N).

### Peak force, minimum force and work

Peak forces at the end of the stretch phase for “STR-30” were significantly greater (89.0 ± 12.0N; *P *< 0.01) compared to the corresponding isometric reference forces (54.5 ± 10.4N) (Table1). In addition, this “STR-30” peak force was not statistically different (*P *= 0.432) to peak forces at the end of the stretch phase in all stretch-shortening tests (91.5 ± 13.7N, 91.4 ± 13.0N and 89.1 ± 13.7N for SSC-30/30, SSC-30/20 and SSC-30/10, respectively). Work during shortening was significantly (*P *< 0.001) lower during pure shortening compared to stretch-shortening, reaching about 60% of the work performed during SSCs for corresponding shortening amplitudes (Table1). In addition, forces were significantly (*P *< 0.001) lower at the end of the shortening phase for the pure shortening compared to the corresponding stretch-shortening cycles (Figs.2, 5).

**Table 1:** Mean work during, and minimum force at the end of pure shortening and stretch-shortening with shortening amplitudes of 30°, 20°, and 10°

Contraction condition	Work [J]						Minimum force [N]					
	30°		20°		10°		30°		20°		10°	
	Mean	SD	Mean	SD	Mean	SD	Mean	SD	Mean	SD	Mean	SD
Pure shortening	1.44	0.23	1.14	0.25	0.70	0.11	8.2	4.0	13.5	5.4	24.4	4.9
Stretch-shortening	2.34	0.36	1.86	0.28	1.10	0.13	15.3	5.4	22.9	6.5	36.7	9.5

All values are significantly (*P *< 0.05) different between contraction conditions at corresponding thumb angle.

### Force after shortening, stretch-shortening and stretch at steady-state

Forces were significantly (*P *< 0.01) depressed to 88.7 ± 12.4%, 84.7 ± 8.3%, and 91.0 ± 10.9% of the isometric reference force following 30°, 20°, and 10° of shortening, respectively (Fig.4). Following 30° of stretching forces were significantly (*P *< 0.01) enhanced to 116.4 ± 7.4% compared to the isometric reference force at a 30° thumb angle. Isometric steady-state forces following stretch-shortening contractions of 30° stretching prior to 30° and 20° shortening (SSC-30/30 and SSC-30/20) did not differ from the purely isometric reference contractions (95.3 ± 8.6% [*P *= 0.052] and 99.7 ± 3.8% [*P *= 0.562]), whereas 30° of stretching followed by 10° shortening (SSC-30/10) resulted in a significantly (*P *< 0.01) enhanced force (110.8 ± 7.4%) relative to the isometric reference force. Forces following stretch-shortening cycles of “SSC-30/20” and “SSC-30/10” were significantly (*P *< 0.01) higher (16.7 ± 8.2% and 22.1 ± 10.0%, respectively) compared to the corresponding isometric forces following 20° and 10° of pure shortening (Fig.4; Table2).

**Table 2:** Mean force 2.5–3 sec after pure shortening, stretch-shortening, pure stretch and isometric reference at corresponding final thumb angle of 0°, 10°, 20°, and 30°

Contraction condition	0°		10°		20°		30°	
	Mean	SD	Mean	SD	Mean	SD	Mean	SD
Isometric [N]	44.0	7.2	49.2	7.7	49.2	7.4	52.0	10.0
Pure Shortening [N]	39.5#	5.8	41.8#	7.8	45.5#	5.9		
Stretch-shortening [N]	42.1	6.6	**48.7**	7.0	**55.4**#	7.7		
Pure Stretch [N]							59.8#	9.6

Bold values indicate significant (*P *< 0.05) difference to pure shortening.

# indicates significant (*P *< 0.05) difference to isometric reference.

**Figure 4:**
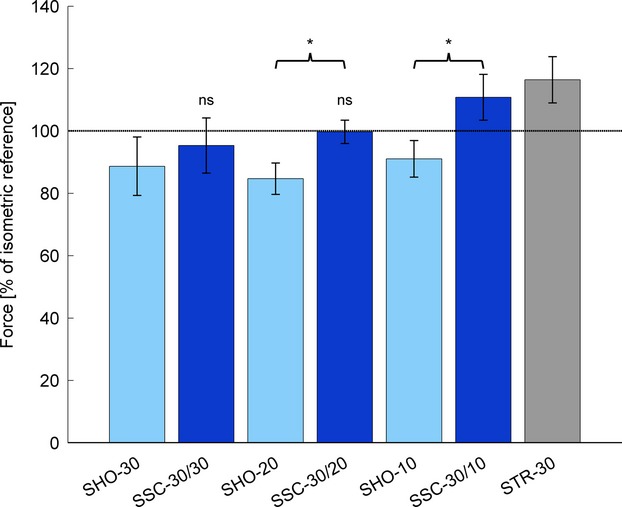
Mean forces and standard deviations, normalized to the forces obtained during purely isometric reference contractions at corresponding thumb angles, 2.5–3 sec after pure shortening (light blue), pure stretch (gray) and stretch-shortening (dark blue) contractions. Pure shortening always started from 30° thumb angle with amplitudes of 30° (SHO-30), 20° (SHO-20) and 10° (SHO-10). Pure stretch and stretch-shortening tests always started from a 0° thumb angle with lengthening of 30°. After lengthening, stretch-shortening trials were immediately followed by 30° (SSC-30/30), 20° (SSC-30/20) or 10° (SSC-30/10) shortening. All forces are significantly different to isometric reference forces (dashed line), except SSC-30/30 and SSC-30/20 (‘ns’). Brackets and asterisks (*) mark significant (*P *< 0.05) differences in forces after stretch-shortening compared to forces after pure shortening.

### Force redevelopment

Using a double exponential function (equation 1), the analyzed data sets (*n* = 80) could be fitted with high accuracy (*R*² ≥ 0.99) (Fig.3). All pooled parameters of force redevelopment were significantly different after the stretch-shortening compared to the pure shortening contractions. Noteworthy, for each shortening amplitude (30°, 20° and 10°) pairwise statistics show that the rate of fast force redevelopment (*k*_*f*_) after SSCs is significantly (*P *< 0.01) higher than after pure shortening tests (Table3). The mean rate of fast force redevelopment across speeds (*k*_*f*_) was 1.7 times higher and the half-life period was 39% shorter after the stretch-shortening compared to the pure shortening contractions (Table3).

**Table 3:** Mean values and SDs of *n* = 10 subjects according to double exponential equation 1 and Half-life periods of force redevelopment after pure shortening (SHO) and stretch-shortening (SSC) contractions. *k*_*f*_ and *k*_*s*_ represent the rate of force redevelopment during the fast and slow recovery-phase for the fast *(f)* and slow *(s)* parts of the force-time traces, respectively

Angle [°]	Condition	*k*_*s*_		*k*_*f*_		Half-life period [msec]	
		Mean	SD	Mean	SD	Mean	SD
0	SHO	0.01	0.03	−6.38	1,18	111	21
	SSC	0.01	0.03	−**9.15**	1,77	88	17
10	SHO	0.02	0.01	−5.48	1,34	132	38
	SSC	**0.00**	0.03	−**10.05**	2,27	83	22
20	SHO	0.02	0.03	−5.10	1,43	138	43
	SSC	−**0.04**	0.03	−**10.09**	2,41	63	14
Pooled SHO		0.02	0.03	−5.65	1,32	127	34
Pooled SSC		−**0.01**	0.03	−**9.76**	2,15	**78**	**18**

Bold values indicate significant (*P *< 0.001) difference between pure shortening and stretch-shortening parameters pairwise at specific angle or pooled.

## Discussion

The main purpose of this study was to determine if mechanisms underlying the phenomenon of residual force enhancement also contribute to the performance enhancement observed in the shortening phase of stretch-shortening cycles. We hypothesized that a) force is enhanced in the steady-state isometric phase after SSCs and/or that b) FD is reduced after SSCs compared to pure shortening contractions of corresponding magnitudes. In contrast to previous studies (Herzog and Leonard 2000; Lee et al. 2001), both hypotheses were confirmed. Active lengthening resulted in enhanced forces throughout the subsequent shortening phase, thereby increasing the mechanical work in the shortening phase of the SSCs. This was accompanied by an increased rate of force redevelopment after SSCs and for the smallest shortening amplitude (10°), a stretch of 30° preceding shortening resulted in a remnant enhanced steady-state isometric force following the SSCs. The enhanced force significantly exceeded the forces observed after pure shortening contractions, as well as the pure isometric forces at corresponding thumb angle. This finding suggests that the RFE generated during the active lengthening phase persisted during the subsequent shortening and isometric phases, thereby contributing to the performance enhancement commonly observed for SSCs.

### Mechanisms of RFE as contributors to enhanced force production during shortening of SSCs

In accordance with most studies on human skeletal muscles (Oskouei and Herzog 2005; Hahn et al. 2010; Power et al. 2013; Seiberl et al. 2013), active lengthening resulted in increased peak forces at the end of stretch as well as in increased posteccentric steady-state forces (i.e., RFE), compared to corresponding isometric references (Fig.4). Importantly, mean peak force reached at the end of the 30° stretch was not different for the pure stretch and the SSC contractions, as one would expect. Since it has been shown that peak forces following active stretching at a given speed and finishing at the same length increase with increasing stretch magnitude (Edman et al. 1982), and since it has been shown that RFE is highly correlated with the force at the end of active lengthening (Bullimore et al. 2007), it is safe to assume that the RFE occurs in the active stretch phase and not the transient force relaxation phase following the stretch. This RFE persisted throughout the entire isometric steady-state phase and might be explained with a Ca^2+^-dependent increase in titin stiffness, the development of half sarcomere nonuniformities, a stretch-induced increase in the number of attached cross bridges, or an increase in the average cross-bridge force (Campbell and Campbell 2011; Edman 2012; Rassier 2012; Herzog 2014). However, in previous studies, the force enhancing effects of active stretching were always abolished by the subsequent shortening, resulting in FD of identical magnitude independent if the shortening was preceded by an active stretch or not (Herzog and Leonard 2000; Lee et al. 2001). In contrast, in our study, FD was always smaller (or even abolished) when active shortening was preceded by an active stretch, while pure shortening contractions of the adductor pollicis always resulted in FD, as expected from the literature (Abbott and Aubert 1952; de Ruiter et al. 1998; Joumaa and Herzog 2010) and irrespective of the final thumb angle or the amplitude of shortening (Fig.4).

The primary mechanism suggested for FD following active shortening is a stress-induced inhibition of cross-bridge attachments in the actin–myosin overlap zone that is newly formed during shortening (Marechal and Plaghki 1979; Lee and Herzog 2009). This stress-induced inhibition has been associated with the amount of mechanical work performed during the shortening phase (Granzier and Pollack 1989; de Ruiter et al. 1998; Herzog et al. 2000). More precisely, it has been shown that FD increases when the product of force and displacement increases, which may result from an increased shortening amplitude or an increased force during shortening. For a given amount of shortening, the newly formed cross-bridge overlap zone should be constant, and the amount of FD would be expected to depend on the amount of actin filament deformation due to the applied force (Herzog et al. 2000). This explanation contradicts our current observations, as the work performed during the pure shortening contractions was smaller than during SSCs but resulted in greater FD. Similarly, Lee et al. (2001) and Herzog and Leonard (2000) found the same FD for pure shortening and SSCs despite vastly different mechanical work in the shortening phase. These observations could be explained by assuming that FD is not only related to the total work produced during shortening, but is also affected by the initial state of the muscle immediately preceding the shortening phase (which may increase the thin filaments resistance to deformation during active shortening). Other factors possibly contributing to the mechanical work during the shortening phase, such as an increase in titin stiffness, would not contribute to FD.

Furthermore, the rate of force redevelopment after shortening contractions was shown to decrease with increasing FD and thereby is sensitive to the amplitude and speed of shortening, ultimately being negatively related to the amount of work performed over a given range of motion (Corr and Herzog 2005). Again, this is in contrast to our findings where a higher work during shortening of SSCs was accompanied by a faster force recovery after depressed states compared to pure shortening. The higher rate of fast force redevelopment *k*_*f*_ (Fig.3), as well as the reduced half-life periods for redeveloped forces (Table3) following stretch-shortening compared to pure shortening contractions support the idea that mechanisms outside actin–myosin interactions contribute to the reduced FD during and following the SCCs. The positive values of the slow component of force redevelopment *k*_*s*_ (Table3) indicate that the force was still slightly increasing and accordingly was not fully recovered for most contractions at the time point of analysis. In contrast, a negative mean *k*_***s***_ was only found for SSCs with 10° shortening amplitude. Here, complete force recovery can be assumed before the time point of analysis and data revealed significantly enhanced forces after stretch-shortening compared to the isometric reference contraction. Hence, besides increased performance due to higher work, SSCs also seem to outperform pure shortening in rapidness of force recovery when the muscle is kept active.

**Figure 5:**
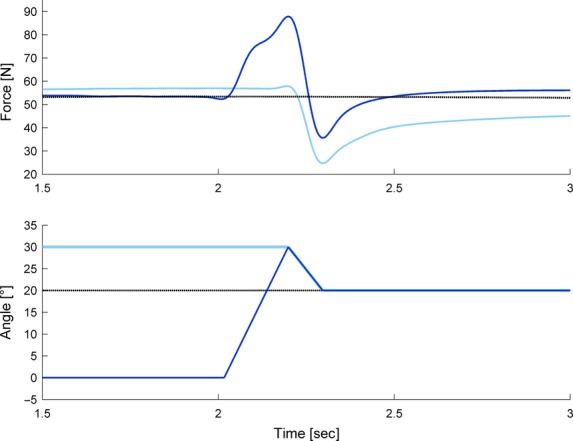
Zoomed-in typical force-time (*n* = 1, filtered with lowpass 10 Hz) and angle-time (reduced schematic illustration) traces of pure shortening (light blue), stretch-shortening (dark blue) and isometric reference contractions (dotted black line) at the final thumb angle position of 20°. Note, following muscle stretching, forces in the stretch-shortening cycles always exceed the forces of the pure shortening contractions and force redevelops to a level that is significantly greater than the isometric reference forces after the pure shortening contractions.

The above explanation does not account for the differences in results between the current study and those reported in the literature (Herzog and Leonard 2000; Lee et al. 2001). In the present study, SSCs always resulted in less FD compared to the corresponding pure shortening tests, despite similar stretch-shortening ratios. Lee et al. (2001) found that the forces during shortening in the SSCs and the pure shortening tests merged halfway through the shortening phase, while this was not observed in the current study (Figs.2, 5). The different findings of Lee et al. (2001) might be explained by the specific speed and magnitude of shortening chosen in that study compared to ours. The speed of stretching and shortening in those previous studies were small compared to those chosen in our study. Since FD is known to be dependent on the shortening speed, whereas RFE is not, the slow speed by Lee et al. (2001) would have produced a relatively great FD which might have offset any remnant effects of the RFE due to the active stretch, while this was not observed here where the speed of shortening was relatively high, and the associated FD small. Furthermore, using a slow speed of shortening increases the time required to complete the shortening phase of the SSC. If the effect of the stretch contraction was time dependent and would disappear in time, the differences in the results by Lee et al. (2001) and those found here might be explained exclusively by the differences in time required for the shortening phase to be completed.

As mentioned above, the force traces in the shortening phase of the Lee et al. (2001) study merged for the SSC and pure concentric contractions about half way through the shortening phase. Since the force at the end of shortening is related to the amount of FD, it is safe to assume that if Lee et al. (2001) had used a shortening magnitude less than half of that actually used (let's say 25% of the original magnitude), their results would also have shown a reduced FD following SSCs compared to the pure shortening contractions, in agreement with the current findings.

### How are these findings situated in classical explanations of performance enhancement during shortening of SSCs?

Several factors have been proposed to contribute to the enhancement of work in the shortening phase of SSCs. These include the storage and release of elastic energy, stretch-induced increased contractility, enhanced activation via reflex activity, and the time required for full muscle activation (van Ingen Schenau et al. 1997b). However, the last factor did not play a role in this study, as muscle activation was always fully established prior to the shortening phase in the pure shortening and the SSCs.

Concerning reflex activity, it is well accepted that stretch reflexes contribute to the enhanced force and work in the shortening phase of SSCs of fast movements, such as hopping (Komi and Gollhofer 1997). Although force in this experiment was electrically evoked, myoelectric force potentiation by recruitment of additional motor units or increased firing rates as a consequence of stretch-reflex responses (Bosco et al. 1981) might have contributed to enhanced forces in the shortening phase of SSCs. However, stretch-reflex-related mechanisms are unlikely to explain the enhanced forces at the end of shortening and during the isometric steady-state force after the SSCs.

A key mechanism thought to explain performance enhancement during the shortening phase of SSCs is the storage and release of elastic energy in series elastic elements located within tendons and sarcomeres (Kubo et al. 1999; Bojsen-Moller et al. 2005). The adductor pollicis was actively lengthened by 30° resulting in elevated passive tension in the muscle-tendon unit. Therefore, storage and subsequent release of elastic energy cannot be ruled out and may have contributed to the observed enhanced work during SSCs, although this proposition is not entirely supported in literature (Chapman and Sanderson 1990; Bobbert et al. 1996; van Ingen Schenau et al. 1997b). However, similar to the reflex activity, storage and release of elastic energy cannot explain the enhanced isometric steady-state forces and reduced FD after SSCs. Hence, the proposed enhancement within the contractile machinery associated with RFE might explain part of the increase in force output during and after the concentric phase of SSC contractions.

Our findings suggest that active lengthening of muscles produced residual force enhancement that was still present in the isometric phase following shortening; that is after “SSC-30/10” experiments. In addition to the higher forces at the beginning of shortening following active muscle stretching compared to isometric contractions, the drop in force toward the end of shortening was less when shortening was preceded by active lengthening rather than isometric contractions. Therefore, the mechanical work during shortening in SSCs was significantly greater than that measured following isometric contractions, as has been shown many times before (Cavagna et al. 1968; Bosco et al. 1987; Gregor et al. 1988). Thus, it appears that RFE contributes to this increased work during shortening in SSCs. However, further research is needed to evaluate the contribution of RFE to this increased work when the speed and magnitude of shortening during SSCs vary, so that the present results might be reconciled with those found earlier (Herzog and Leonard 2000; Lee et al. 2001) that did not show an effect on FD when relative slow shortening contractions were preceded by active muscle stretching.

### Limitations of experimental setup and thumb model

The electrically stimulated m. adductor pollicis is a well-established model to investigate in vivo human muscle function noninvasively (Lee and Herzog 2003; de Ruiter and de Haan 2003; Oskouei and Herzog 2005). However, there are limitations of this approach that need to be considered when evaluating the results of our study.

The thumb dynamometer kinematics were controlled for acceleration of 500°s^−2^. Due to the different shortening amplitudes, this approach resulted in different mean shortening velocities, increasing with increasing range of motion. Although this kinematical setup may be adequate to model in vivo SSCs, where isovelocity is unlikely to occur, for experimental testing the interpretation of FD in relation to (stretch-) shortening amplitude is confounded by the different shortening velocities and amplitudes which are known to be related to the amount of FD (de Ruiter et al. 1998). Therefore, comparisons of forces across different shortening amplitudes need to be performed with caution.

History dependence of muscle action depends on significant length changes of muscles, fibers and sarcomeres. Although the external thumb adduction angle was controlled, we did not directly measure muscle architectural changes during stretch, shortening, and stretch-shortening cycles. Hence, actual muscle length changes caused by the thumb angular displacements are unknown. However, our results of the pure shortening and pure stretch contractions are in accordance with previous work (Lee and Herzog 2002, 2003; Oskouei and Herzog 2005), suggesting that muscle length changes were also sufficient to cause history-dependent effects in stretch-shortening contractions of m. adductor pollicis.

## Conclusion

Based on the results of this study, we conclude that RFE acquired during stretch contributes to the increased force and mechanical work observed in the shortening phase of SSCs, and can abolish FD following SSCs. Therefore, RFE might be the missing link that, in addition to the activation dynamics, stretch-reflex responses, and the release of stored elastic energy, may explain the increase in force/work observed in the shortening phase of SSCs. In view of previously published results where RFE was abolished during the shortening phase of SSCs (Herzog and Leonard 2000; Lee et al. 2001), it appears that RFE acquired during active muscle stretching is abolished in a transient manner during shortening. At this point, it is not clear if this transient disappearance depends on the speed and magnitude of shortening or is merely a function of time. Within the context of the present findings, the mechanisms of RFE clearly contribute to elevated SSC performance.
